# Utilization Practices of Inferior Vena Cava Filters at an Academic Medical Center

**DOI:** 10.7759/cureus.55505

**Published:** 2024-03-04

**Authors:** Joud El Dick, Palak Shah, Asit Kr Paul

**Affiliations:** 1 Department of Internal Medicine, Virginia Commonwealth University Medical Center, Richmond, USA; 2 Department of Internal Medicine, Division of Hematology, Oncology and Palliative Care, Virginia Commonwealth University Medical Center, Richmond, USA

**Keywords:** ivc filter retrieval, inferior vena cava filter, ivc filter complication, device removal, pulmonary embolism prevention, retrospective studies

## Abstract

Introduction: Anticoagulation is the mainstay of management for patients with venous thromboembolism (VTE), which includes deep vein thrombosis (DVT) and pulmonary embolism (PE). Inferior vena cava (IVC) filters are indicated in select patients who are not candidates for anticoagulation. There is a lack of quality evidence supporting other indications. In addition, long-term benefits and safety profiles of IVC filters have not been established. We investigated the utilization practice of IVC filters in a contemporary series of patients in a tertiary academic medical center.

Methodology: A retrospective review of 200 patients who received IVC filters at Virginia Commonwealth University (VCU) Medical Center in the years 2017 and 2018 was conducted. Adult patients 18 years of age or older with or without cancer were included, and patients were selected consecutively until data on 200 patients were collected. Data on patient demographics, an indication of IVC filter placement, filter retrieval rate, and re-thrombosis events over a median follow-up period of nine months were extracted from the electronic medical record and analyzed.

Results: A total of 200 patients (105 male and 95 female) were included with a median age of 61 years (range 17-92 years). Of the 200 patients, 97 (48.5%) had a DVT, 28 (14%) had a PE, 73 (36.5%) had both a PE and DVT, and 2 (1%) had thrombosis at other sites. A total of 130 (65%) patients had an IVC filter placed because of a contraindication to anticoagulation, while 70 (35%) had an IVC filter placed for other nonstandard indications, which included new or worsening VTE despite anticoagulation, recent VTE who must have anticoagulation held during surgery, primary prevention in high-risk patients, and extensive disease burden among other reasons. Seventy-two (36%) patients had active malignancy at the time of filter placement, and 64 (32%) were lost to follow-up. Of the 119 patients who were potentially eligible for filter retrieval, 55 (46%) patients had their IVC filters removed at a median of five months after insertion. Of the 55 patients who had IVC filters removed, 8 (14.5%) patients experienced a re-thrombosis event within a median follow-up of 39 months. Of the 145 patients who still had their filter in place at the time of death or last follow-up, 5 (3.4%) patients experienced a re-thrombosis event within a median follow-up of three months.

Conclusions: One-third of the patients in this series had an IVC filter placed without a standard indication, and less than half of them had the IVC filters removed within one year of placement. Additionally, one-third of the patients were lost to follow-up, highlighting the need for improved structured follow-up programs and education among both patients and providers regarding the indications for placement and retrieval to minimize complications.

## Introduction

Venous thromboembolism (VTE), which includes deep vein thrombosis (DVT) and pulmonary embolism (PE), is a significant cause of morbidity and mortality [1]. VTE is the leading cause of preventable in-hospital mortality and accounts for 300,000 deaths annually in the United States [2]. Inferior vena cava (IVC) filters are widely used in the United States, with approximately 96,000 IVC filters placed in 2014 [3]. The mainstay of treatment for patients with VTE is systemic anticoagulation. However, for patients with a contraindication to anticoagulation, IVC filter placement is an option. Several major professional groups, including the American College of Chest Physicians and the Society of Interventional Radiology, have published guidelines for the use of IVC filters. These guidelines agree that IVC filters should be placed in most patients with VTE and a contraindication to anticoagulation [1,4]. However, there is no consensus among the guidelines on other possible indications, such as VTE despite anticoagulation, patients with recent VTE who must have anticoagulation held for surgery, and IVC placement for primary prevention in high-risk patients [5]. Moreover, as more filters have been placed, complications such as subsequent DVT, worsening post-thrombotic syndrome, as well as migration, perforation, and thrombosis of an IVC filter have been recognized [6]. Overall, there are unanswered questions regarding their efficacy for non-standard indications and more widely recognized complications. In this study, we investigated the utilization practice of IVC filters at a tertiary academic medical center, Virginia Commonwealth University (VCU) Medical Center. We evaluated whether IVC filters were placed based on evidence-based indications and assessed outcomes, retrieval rates, post-placement follow-up, and complication rates.

## Materials and methods

Study settings and inclusion and exclusion criteria

This was a retrospective chart review of patients who had IVC filters placed at VCU Medical Center in Richmond, Virginia. IVC filters were placed by interventional radiologists and vascular surgeons from January 2017 to December 2018. The study included data from adult patients aged 18 years and older. We excluded patients who did not have their IVC filters placed at VCU Medical Center even if their filter was removed at our institution. The study was approved by the VCU Institutional Review Board (study ID HM20022416).

Data collection

Medical record numbers (MRNs) of patients who had IVC filters placed during the specified period were obtained from the electronic medical record (Cerner, Kansas City, MO) through the radiology department using Discern Analytics 2’s advanced filtering capabilities to identify eligible patients, which is a Cerner analytics platform. The platform utilized IVC filter brand names, as well as the values and codes “IVC filter placement,” “IVC filter removal,” and “IVC filter repositioning” to identify the relevant MRNs. To ensure accuracy, duplicate MRNs were eliminated and a subsequent manual review of electronic charts was performed to confirm that the included patients met the predefined inclusion criteria. Data on 200 patients was available for analysis. Basic demographic information, including age at IVC filter insertion, race, sex, comorbidities, and status of active malignancy at the time of filter insertion were identified. In addition, the type of thrombosis, indication for IVC filter placement, type of filter placed, follow-up rate, and retrieval rate were collected. Data was entered into Redcap where it was subsequently deidentified using a unique patient identifier.

Data analysis and variables

Data were extracted from Redcap and analyzed using Statistical Package for Social Sciences (SPSS) version 27.0 (IBM Corp, Armonk, NY). Descriptive statistics, including frequencies for all categorical variables, median age, median follow-up time, time to IVC filter retrieval, and complication rates, were calculated.

## Results

Among 200 patients analyzed, there was a female-predominant distribution, with 105 (52.5%) females and 95 (47.5%) males. The median age at IVC filter insertion was 61 years. Most patients, comprising 102 (51%) White individuals, 89 (44.5%) Black individuals, and 9 (4.5%) Hispanic or individuals of other races. A total of 194 (97%) patients had retrievable IVC filters placed, while 6 (3%) had permanent filters placed. Seventy-two (36%) patients had a diagnosis of cancer not in remission at the time of IVC filter insertion. Demographics and the presence of cancer at the time of filter insertion are outlined in Table 1. Among the 72 patients with cancer who received IVC filters, the most common type of cancer was gastrointestinal cancer in 15 (20.8%) patients. The most frequent comorbidities among all 200 patients with IVC filters were hypertension in 103 (51.5%) patients, obesity in 79 (39.5%) patients, diabetes in 47 (23.5%) patients, and tobacco use in 35 (17.5%) patients. The type and frequency of malignancy, as well as past medical history, are further summarized in Tables 2-3, respectively.

Of all 200 patients, 97 (48.5%) had an IVC filter placed for DVT alone, 73 (36.5%) for PE and DVT, 28 (14%) for PE alone, and 2 (1%) for thrombosis at a different site. Table 4 summarizes the frequency and type of thrombosis. A total of 130 (65%) patients had an IVC filter placed in the setting of a clear contraindication to anticoagulation, which included patients with intracranial hemorrhage or active gastrointestinal bleeding as the most common contraindications, as detailed in Table 5. The remaining 70 (35%) patients had IVC filters placed for other nonstandard indications, which included new or worsening VTE despite anticoagulation, recent VTE with anticoagulation held in the perioperative period for surgery other than intracranial or spinal neurosurgery, primary prevention in high-risk patients, extensive disease burden, anticoagulation nonadherence, and other reasons such as a remote history of intracranial or gastrointestinal bleeding, as well as high-fall risk. Further breakdown of the indications for IVC filter placement is summarized in Table 5.

**Table 1:** Basic characteristics.

Basic characteristics	Number (%), *N* = 200
Median age at filter insertion	61 years (range 17-92 years)
Gender	
Female	105 (52.5%)
Male	95 (47.5%)
Race/Ethnicity	
White	102 (51%)
Black	89 (44.5%)
Hispanic	1 (0.5%)
Other/Not known	8 (4%)
Malignancy at the time of filter insertion	
Yes	72 (36%)
No	128 (64%)

**Table 2:** Types of malignancy.

Malignancy type	Number (%), *N *= 72
Gastrointestinal cancer	15 (20.8%)
Non-small cell lung cancer	9 (12.5%)
Acute myeloid leukemia	6 (8.3%)
Glioblastoma	5 (7%)
Head and neck cancer	4 (5.6%)
Prostate cancer	4 (5.6%)
Other	29 (40.3%)

**Table 3:** Medical history.

Disease	Number (%), *N *= 200
Hypertension	103 (51.5%)
Obesity	79 (39.5%)
Diabetes mellitus	47 (23.5%)
Tobacco use	35 (17.5%)
Heart failure	29 (14.5%)
Previous history of thrombosis	28 (14%)
Personal history of cancer	18 (9%)
Obstructive sleep apnea	12 (6%)
Coronary artery disease	11 (5.5%)
Atrial fibrillation	6 (3%)
Peripheral vascular disease	4 (2%)
Hypercoagulable disorder	3 (1.5%)

**Table 4:** Types of thrombosis. DVT, deep vein thrombosis; PE, pulmonary embolus; IVC, inferior vena cava

Type of thrombosis	Number (%), *N *= 200
DVT only	97 (48.5%)
PE only	28 (14%)
PE and DVT	73 (36.5%)
IVC thrombus	1 (0.5%)
Renal vein thrombus	1 (0.5%)

**Table 5:** Indication for IVC filter placement. IVC, inferior vena cava; VTE, venous thromboembolism; GI, gastrointestinal; CVA, cerebral vascular accident; CNS, central nervous system; HIPEC, hyperthermic intraperitoneal chemotherapy; DVT, deep vein thrombosis

Indication for IVC filter placement	Number (%), *N *= 200	Further breakdown
Evidence-based: VTE and a contraindication to anticoagulation	130 (65%)	Intracranial hemorrhage: *n *= 31; active GI bleed: *n *= 19; thrombocytopenia (<50,000/microL): *n *= 11; recent neurosurgery (intracranial or spinal) who must have anticoagulation held postoperatively: *n *= 7; hematuria: *n *= 6; recent ischemic or hemorrhagic CVA who must have anticoagulation held poststroke or post-thrombectomy: *n *= 6; intracranial malignancy with hemorrhage: *n *= 5; anemia requiring transfusions: *n *= 5; other bleeding (retroperitoneal hematoma, hemoptysis, hemothorax, and extremity hematoma): *n *= 40.
Recent VTE who must have anticoagulation held or surgery other than intracranial or spinal neurosurgery	18 (9%)	N/A
New or worsening VTE despite anticoagulation	13 (6.5%)	N/A
Thrombosis with extensive disease burden and no contraindication to anticoagulation	10 (5%)	N/A
IVC placement for primary prevention in high-risk patients	2 (1%)	N/A
Other	27 (13.5%)	Remote history of GI bleed or CNS hemorrhage (>3 months): *n* = 6; high-fall risk: *n *= 5; intracranial malignancy without hemorrhage: *n *= 3; noncompliance with anticoagulation: *n *= 1; anticoagulation being held due to upcoming HIPEC: *n *= 1; recent aortic root and aortic valve replacement: *n *= 1; chronic DVT in the setting of intracranial hemorrhage: *n *= 1; post-hepatic artery embolization: *n *= 1; no clear identified reason: *n *= 8

Of 200 patients, 55 (27.5%) had their filters removed at a median of five months after insertion (range of four days to 84 months), with 11 patients having their filters removed within four months of placement. Of the 55 patients who had their IVC filters removed, two patients expired or were enrolled in hospice care at the time of the last follow-up. The remaining 145 (72.5%) patients had their IVC filter still in place at the time of death or last follow-up. Of these 145 patients, 64 (44.1%) patients were lost to follow-up regarding assessment for IVC filter removal, and the remaining 81 (55.9%) patients were not eligible for removal at three months post-placement due to ongoing contraindication to anticoagulation, death, enrollment in hospice at the time of the last follow-up, or due to initial placement of a permanent filter. Of the 72 patients with active malignancy who received IVC filters, 45 (62.5%) had expired or enrolled in hospice at the time of the last follow-up.

Of the 55 patients who had their IVC filters removed, 10 (18.2%) patients experienced one or more complications, with 8 experiencing a re-thrombosis event. The location of re-thrombosis included IVC filter thrombus, IVC thrombus extending above or below the IVC filter, or lower extremity DVT. The remaining complications experienced were filter migration, tilting, bacteremic seeding of the filter, and stenosis at the level of the filter. Of the 145 patients whose filter was still in place at the time of death or last follow-up, 6 (4.1%) patients experienced a complication event - 5 patients with re-thrombosis and 1 patient with filter fracture. There was a higher median follow-up of 39 months in patients who had their IVC filters removed as opposed to three months in patients who did not have their IVC removed at the time of death or last follow-up.

## Discussion

In this series, 70 (35%) patients had IVC filters placed for nonstandard indications or relative contraindications. Some IVC filters, for example, were placed in patients with a remote history of gastrointestinal bleeding or central nervous system hemorrhage that occurred more than three months before filter placement, with no active evidence of ongoing hemorrhage. Other filters were placed in patients with high-fall risk where anticoagulation would not have been contraindicated. Our data suggest that variability exists among providers regarding the placement of IVC filters in patients with relative contraindications.

Moreover, of the 119 patients who were potentially eligible for filter retrieval, 55 patients had their IVC filters removed, with an overall retrieval rate of 46.2% and a median retrieval time of five months after insertion. A total of 64 (32%) patients were lost to follow-up regarding assessment for IVC filter removal. As for complication rates, 16 (8%) patients in total had experienced an IVC-related complication event, with re-thrombosis being the most frequent. There seemed to be a higher number of complication events in patients who had their IVC filters removed, but this is likely confounded by the fact that these patients were seen in the clinic for removal and not lost to follow-up. In addition, most of the complications noted were documented during the retrieval procedure itself, and the lower median follow-up time of three months in patients who did not have their IVC filters removed, due to loss to follow-up and death, as opposed to 39 months in patients who had their IVC filters removed, likely lead to the detection of fewer complication events.

The Society of Interventional Radiology recommends the use of structured follow-up programs to increase IVC filter retrieval rates and detect complications [1]. Several methods, described in the literature, included the implementation of an IVC filter follow-up clinic with a multidisciplinary team approach, the use of patient registries, the implementation of patient education and IVC filter coordinator, and computerized reminder systems [7]. In our institution, after IVC filter placement, an automated outpatient appointment with interventional radiology was scheduled to assess for continued need for an IVC filter.

In one study, retrieval rates in major academic centers improved from 11% pre-implementation to 54% post-implementation, while another study reported an increase from 23% to 45% [8,9]. These rates were comparable to the overall retrieval rate in our cohort. Other studies showed even higher retrieval rates. Schuchardt et al. found that with the implementation of an IVC filter retrieval clinic, 62.9% of the IVC filters that were potentially retrievable were removed during the study period. The process consisted of quarterly follow-up during 12 months and enrollment of patients after they had their IVC filter placed [10]. Another study by Sutphin et al. showed a 52% retrieval rate with automated clinic scheduling [11]. Inagaki et al. found that with the establishment of a secure IVC database, retrieval rates improved from 52.9% to 72.9%. Retrieval decisions were first made 90 days after insertion, and an alert message would appear within the database if a patient lacked a documented plan after this follow-up period [8].

Mohapatra et al. found, however, that despite the improvements in major academic centers, the annual retrieval rate continued to be low at 6.6% in the general IVC filter population [12]. Overall, there is a growing body of literature supporting the need for a robust and structured IVC filter retrieval program to identify patients who no longer indicate an IVC filter. There is also a need for increased education among providers and patients regarding the indications for removal of IVC filters once the contraindication for anticoagulation has resolved and anticoagulation has been resumed, or when protection from pulmonary embolism is no longer needed. Primary care providers and specialists should feel confident in placing referrals for IVC filter removal assessments once the need is recognized to improve retrieval rates and minimize complications.

Limitations

As with any study conducted through retrospective chart review, ours inherently faced limitations, including unknown confounding variables. Additionally, there was incomplete follow-up data due to the significant number of patients lost to follow-up. This included patients with IVC filters placed at our institution who may have had them removed at different institutions, potentially underestimating follow-up and retrieval rates. Furthermore, we excluded IVC filters initially placed at other institutions but removed by our interventional radiology department. Inconsistent recording practices and inadequate documentation regarding the indication for filter placement further limited the study. Finally, being a single-institution study, our results may not be generalizable to the national or international population.

## Conclusions

One-third of the patients in this series had an IVC filter placed without a standard indication, and many patients with terminal conditions received IVC filters. Furthermore, less than half of them had the IVC filters removed within one year of placement, and one-third of the patients were lost to follow-up for IVC filter removal assessment. These findings highlight the need for improved structured follow-up programs and education among both patients and providers regarding the indications for placement and retrieval to minimize complications.
